# Seasonal variation in wing geometry of the malaria vector *Anopheles maculatus* (Diptera: *Culicidae*) in Western Thailand

**DOI:** 10.5455/javar.2025.l949

**Published:** 2025-09-08

**Authors:** Suchada Sumruayphol, Tanawat Chaiphongpachara, Sedthapong Laojun

**Affiliations:** 1 Department of Medical Entomology, Faculty of Tropical Medicine, Mahidol University, Bangkok, Thailand; 2 Department of Public Health and Health Promotion, College of Allied Health Sciences, Suan Sunandha Rajabhat University, Bangkok, Thailand

**Keywords:** Malaria vector, wing size, wing shape, geometric morphometrics, landmark-based geometric morphometrics, outline-based geometric morphometrics

## Abstract

**Objective:**

*Anopheles maculatus *is recognized as an important malaria vector in Thailand and other countries within the Greater Mekong Subregion. This study employed both landmark- and outline-based geometric morphometrics (GM) approaches to assess seasonal variation in the wing structure and wing contour of *A*. *maculatus* from malaria hotspots in western Thailand across three seasons: hot, wet, and dry.

**Materials and Methods:**

We analyzed seasonal variation in wing structure and contour using landmark-based and outline-based GM approaches, respectively, applied to the same image set of wing samples. Statistical differences in size and shape among seasonal populations were evaluated using a non-parametric analysis of variance (1,000 replicates), followed by a Bonferroni post hoc test. A *p*-value of less than 0.05 was used as the criterion for statistical significance in all analyses.

**Results:**

The size analyses revealed a significant difference in wing structure between the hot and dry seasons (*p *< 0.05), while no significant differences (*p* > 0.05) in wing contour across seasonal populations were detected. Significant differences (*p* < 0.05) in wing structure based on shape were detected between *A*. *maculatus *populations in the dry and hot seasons, as well as between populations in the dry and wet seasons. Wing contour analysis based on shape showed a significant difference (*p* < 0.05) only between the populations from the dry and wet seasons.

**Conclusion:**

These findings provide us with valuable information about the seasonal adaptation of *A*.* maculatus*, thus enhancing our understanding of vector population dynamics and potentially improving malaria surveillance strategies.

## Introduction

Malaria remains a significant global health challenge, especially in tropical and subtropical regions [1,2]. In 2023, it was estimated that there were 263 million malaria cases and 597,000 deaths across 83 countries [3]. This severe disease is caused by protozoan parasites in the genus *Plasmodium*, which encompasses over 200 species. Of these, five species—*Plasmodium falciparum, Plasmodium vivax, Plasmodium malariae, Plasmodium ovale,* and *Plasmodium knowlesi *—are known to infect humans [3]. Female *Anopheles *mosquitoes (Diptera: *Culicidae*) have been proven to be vectors of the malaria parasites. Currently, there are 512 formally recognized *Anopheles *species [4]. However, not all *Anopheles* species are vectors; the ability of each species to transmit the disease depends on its behavior and capacity. Of the total, approximately 80 *Anopheles *species are known to transmit malaria parasites, and about 40 of these are considered primary vectors of the parasites [2,5].

In Thailand, malaria continues to pose a significant public health concern, particularly along its borders with Myanmar, Malaysia, Laos, and Cambodia [1,2,6]. Despite an annual decrease in cases, 14,684 were still reported in 2024, underscoring the ongoing challenge [7]. Tak province, located in western Thailand, recorded the highest incidence with 6,432 cases [7]. These data highlight the persistent prevalence of malaria, especially in Tak province, and emphasize the urgent need for effective management strategies to control the spread of the disease in these regions. Furthermore, understanding the biology of malaria vector mosquitoes in the region is crucial for effective disease surveillance [8].

More than 74 species of *Anopheles *mosquitoes have been recorded in Thailand. Each species exhibits distinct epidemiological traits, habitat preferences, breeding sites, biting behaviors, levels of insecticide resistance, and vectorial capacities [9]. These differences directly influence their roles as malaria vectors. The most important malaria-­transmitting species in Thailand belong to the subgenus *Cellia*, which includes members of the Leucosphyrus group (series: Neomyzomyia), the *Maculatus* group (series: Neocellia), and the Minimus subgroup (series: Myzomyia) [10]. Members of the *Maculatus* group are widely distributed across the Indian subcontinent, Southeast Asia, and as far as Taiwan [11]. This group comprises nine recognized species: *Anopheles sawadwongporni, Anopheles maculatus, Anopheles dravidicus, Anopheles* *greeni, Anopheles notanandai, Anopheles* *willmori, Anopheles* *pseudowillmori, Anopheles dispar*, and *Anopheles rampae*. In addition, genetic evidence of another sibling species in the Maculatus group, known as the “Javanese form,” has been reported from Java, Indonesia, by Ali et al. [12]. Of these, seven species have been reported in Thailand, with *A*. *maculatus* recognized as the most significant malaria vector in the group. *Anopheles *maculatus is commonly found in forested border areas and is frequently associated with malaria outbreaks [13].

The assessment of intraspecific variation in mosquitoes is crucial and highly beneficial for understanding how environmental pressures influence disease vectors [14,15]. Such variation, driven by environmental factors, can occur at both the genetic and morphological levels, and modern techniques have greatly enhanced our ability to detect these differences. For instance, molecular approaches are commonly employed to detect genetic variation [16,17], while morphometric tools are used to examine morphological variation [18]. Research into intraspecific variation often entails comparing the same mosquito species in various environments to assess how environmental factors influence mosquito morphology [19]. Abiotic factors such as elevation, relative humidity, rainfall, latitude, temperature, and ecoregion significantly affect mosquito morphology, particularly the size and shape of the wings [20,21]. However, the extent of these effects varies depending on the mosquito species studied. Understanding these relationships is essential for interpreting vector behavior and developing more effective vector control strategies. Despite its importance, no studies to date have examined seasonal variation in wing morphology of primary malaria vectors in endemic areas of Thailand.

Geometric morphometrics (GM) is a modern approach for analyzing differences in shape and size [22]. It has been widely applied in mosquito research for various purposes, such as examining intra- and interspecific variation, identifying sexual dimorphism, studying plasticity and deviation, distinguishing laboratory strains, and detecting parasites [20]. The mosquito wing, with its two-­dimensional structure and well-defined shape, serves as an ideal organ for this type of analysis, helping to minimize digitizing errors [20]. There are two primary GM methods used in mosquito identification: the landmark-based approach and the outline-based approach. The landmark-based method relies on anatomical landmarks to measure size and shape, whereas the outline-based method uses pseudo-landmarks to represent contours [23]. The main advantages of these GM techniques are their low cost, minimal equipment requirements, and rapid data analysis [20]. These strengths highlight the potential of GM methods for investigating seasonal variation in phenotypic plasticity in *A*. *maculatus*.

Based on the information and challenges described, this study aimed to evaluate the seasonal variation in the wing structure of *A*. *maculatus* from malaria hotspots in western Thailand using a landmark-based GM approach and to assess variation in wing contour using an outline-based GM approach. The primary research question addressed was: Does the wing structure and outline of *A*.* maculatus* vary significantly across different seasons in western Thailand? The findings from this study enhance our understanding of the physiological adaptations of this mosquito vector in response to seasonal environmental changes, which will aid in the more effective monitoring of vector population dynamics.

## Materials and Methods

### Ethical approval

Ethical approval for this study was granted by the Animal Care and Use Committee of the Faculty of Tropical Medicine, Mahidol University, Bangkok, Thailand, under the EC number 033-2018.

### Mosquito collection and species identification

**Figure 1.**
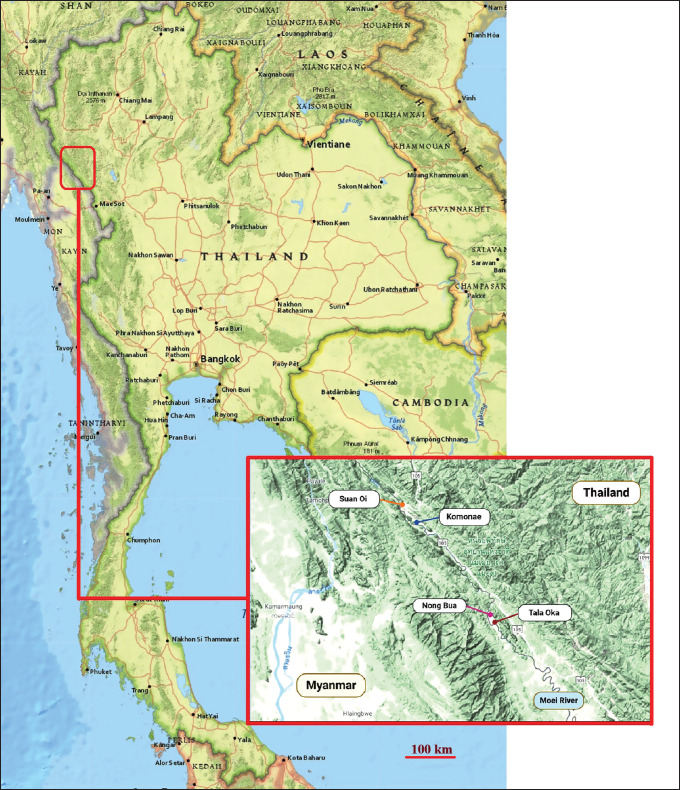
Four malaria hotspot villages along the Thai-Myanmar border, where *A. maculatus* was collected for this study: Suan Oi (17°33’36.5” N, 97°55’12.5” E); Komonae (17°31’57.4” N, 97°56’57.9” E); Nong Bua (17°20’24.8” N, 98°06’24.6” E); and Tala Oka (17°19’24.5” N, 98°06’58.0” E). The map images were sourced and modified from the USGS National Map Viewer (https://apps.nationalmap.gov/3depdem/) and Google Earth Pro (version 7.3.6.10201).

*Anopheles* mosquitoes were collected from four malaria hotspot villages along the Thai-Myanmar border [24]: Suan Oi (17°33’36.5” N, 97°55’12.5” E), Komonae (17°31’57.4” N, 97°56’57.9” E), Nong Bua (17°20’24.8” N, 98°06’24.6” E), and Tala Oka (17°19’24.5” N, 98°06’58.0” E) (Fig. 1). These villages are located in the Tha Song Yang District of Tak Province, western Thailand. Thirty CDC light traps (BiQuip model 2836BQ, USA) were employed for mosquito collection, operating nightly from 6 p.m. to 6 a.m. In each village, the traps were evenly distributed, with 15 placed indoors and 15 outdoors at 30 houses reporting malaria cases. The selected houses were used for mosquito trapping during every sampling round without any changes. Indoor traps were hung approximately 1.5 m above the ground in living rooms or within the single room in one-room houses. Outdoor traps were installed at the same height, approximately 20 m from the houses. Mosquito sampling was conducted for 1 week each month, typically in the middle of the month, from January to December 2015. All captured *Anopheles* specimens were euthanized via cryotherapy and sent to the Department of Medical Entomology, Faculty of Tropical Medicine, Mahidol University, Bangkok, Thailand, for species identification.

Seasonal classification in the study followed a previous report, which defined three distinct seasons: the hot season (March to May), the wet season (June to August), and the dry season (September to February) [24]. Seasonal meteorological data for the study sites in 2015 are presented in Table 1. The hot season features the highest temperatures of the year, accompanied by low to moderate rainfall and relatively low humidity. In contrast, the dry season is cooler, with the lowest average temperatures, minimal rainfall, and moderate humidity. While both the hot and dry seasons experience limited rainfall, the primary distinction is temperature—higher during the hot season and lower during the dry season, which also boasts more stable atmospheric conditions. The wet season is characterized by heavy rainfall, high humidity, and slightly lower temperatures compared to the hot season.

For the morphological identification of mosquito species, specimens with damaged or missing body parts, which hinder accurate morphological analysis, were excluded from verification. The remaining complete samples were subsequently examined under a Nikon AZ 100 M stereoscope (Nikon Corp., Tokyo, Japan) and identified by using the Illustrated Keys to the Mosquitoes of Thailand [25]. Two hundred intact and unbroken wings of *A*. *maculatus*, divided among 20 individuals from the hot season, 99 from the wet season, and 81 from the dry season, were used to assess seasonal variation in wing structure and contour using landmark-based and outline-based GM analyses, respectively.

### Wing preparation

After morphological identification, the right wings of *A*. *maculatus* were meticulously dissected from the thoraxes by using forceps and needles. To prevent inaccuracies in the GM analysis, damaged wings were discarded. For optimal visibility of the wing veins during the plotting of landmark coordinates, wing scales were carefully removed with a needle under a Nikon AZ 100 M stereoscope. Wings were mounted onto microscope slides using Hoyer’s medium as the embedding solution. The prepared wings were covered with another glass slide and dried at room temperature for about 7 days. Once the slides were fully dried, each wing was imaged at 40x magnification using a Nikon DS-Ri1 SIGHT digital camera mounted on a stereomicroscope. To maintain measurement accuracy, a 1 mm scale bar was included in the lower-left corner of every image.

### Landmark and outline digitizations

**Figure 2.**
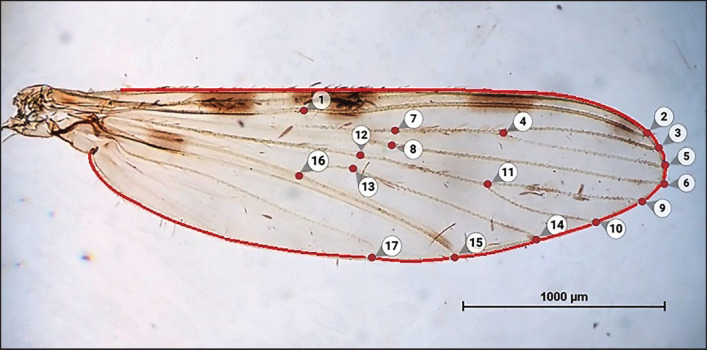
Seventeen landmarks on the wing of *Anopheles maculatus*, which were used for landmark-based GM analysis, and the contour (red outline) used for outline-based GM analysis.

We analyzed seasonal variation in wing structure and wing contour using the landmark-based and outline-based GM approaches, respectively. We applied both approaches to the same set of samples. To assess the wing structure, undamaged right wing images were used to digitize anatomical landmarks at the intersections of the wing veins in samples from each seasonal collection. Seventeen clearly visible landmarks were selected for accurate plotting (Fig. 2). For the outline-based analysis, the same right-wing images were used to digitize pseudo-landmarks along the wing contour (Fig. 2).

### Repeatability test

To assess the precision of the digitization process, a random sample of 20 wings from each season was selected and digitized twice by the same operator. The measurement errors for landmark and outline digitizations were assessed using the repeatability index, calculated through the Procrustes analysis of variance (ANOVA) method [26].

### Wing size analysis

Wing size in the landmark-based GM approach was quantified using centroid size (CS), which represents the square root of the total squared distances from each landmark to the geometric center of the entire landmark set. In the outline-based GM approach to wing contour analysis, size was determined by measuring the length of the perimeter. Statistical differences in size among seasonal populations were assessed using a non-parametric ANOVA (1,000 replicates), followed by a Bonferroni post-hoc test. All statistical analyses were conducted using a significance level of *p* < 0.05 to determine meaningful differences.

**Table 1.** Seasonal meteorological data for 2015 averaged over four study sites during three seasons. Raw data obtained from the Mae Sot weather station in Thailand.

Meteorological parameters	Hot season(March to May)		Wet season(June to August)		Dry season (September to February)	
	Average	SD	Average	SD	Average	SD
Temperature (°C)	28.87	0.85	26.63	0.75	25.37	1.87
Total rainfall (mm)	56.07	44.08	328.63	123.68	68.18	83.59
Relative humidity (%)	68.67	6.35	87.67	2.31	75.17	8.45

SD = standard deviation.

### Wing shape analysis

Wing shape in the landmark-based GM analysis was characterized by aligning all landmark configurations using Generalized Procrustes Analysis, after which principal component analysis (PCA) was performed to extract the key shape descriptors used in subsequent analyses. In contrast, the outline-based GM approach applies elliptic Fourier analysis to derive shape descriptors from wing contours. These descriptors, expressed as normalized elliptic Fourier coefficients (NEF), were subjected to PCA, and the resulting principal components served as the definitive variables for shape characterization.

The final shape variables were used as inputs for discriminant analysis (DA) to assess shape variation among seasonal populations. Variation among these groups was visualized using factor maps based on the first (DF1) and second (DF2) discriminant factors. Mahalanobis distances, which measure the similarity between groups, were calculated from the DA. Statistical differences in shape among seasonal populations were assessed using a non-parametric ANOVA (1,000 replicates), followed by a Bonferroni post hoc test. The reliability of group assignments was evaluated using a cross-validation procedure to assess classification accuracy. In this process, individual samples were iteratively removed, and their assignations recalculated based on the analysis of the remaining data. Additionally, classification trees were constructed using a single-linkage hierarchical classification algorithm to depict the shape similarities between groups.

### Software

The GM analyses, including landmark and contour digitization, repeatability testing, wing size analysis, wing shape analysis, and validated classification testing, were conducted using the XY online morphometrics (XYOM) tool [27]. This software is accessible at https://xyom.io.

## Results

### Seasonal variation in wing structure

**Figure 3.**
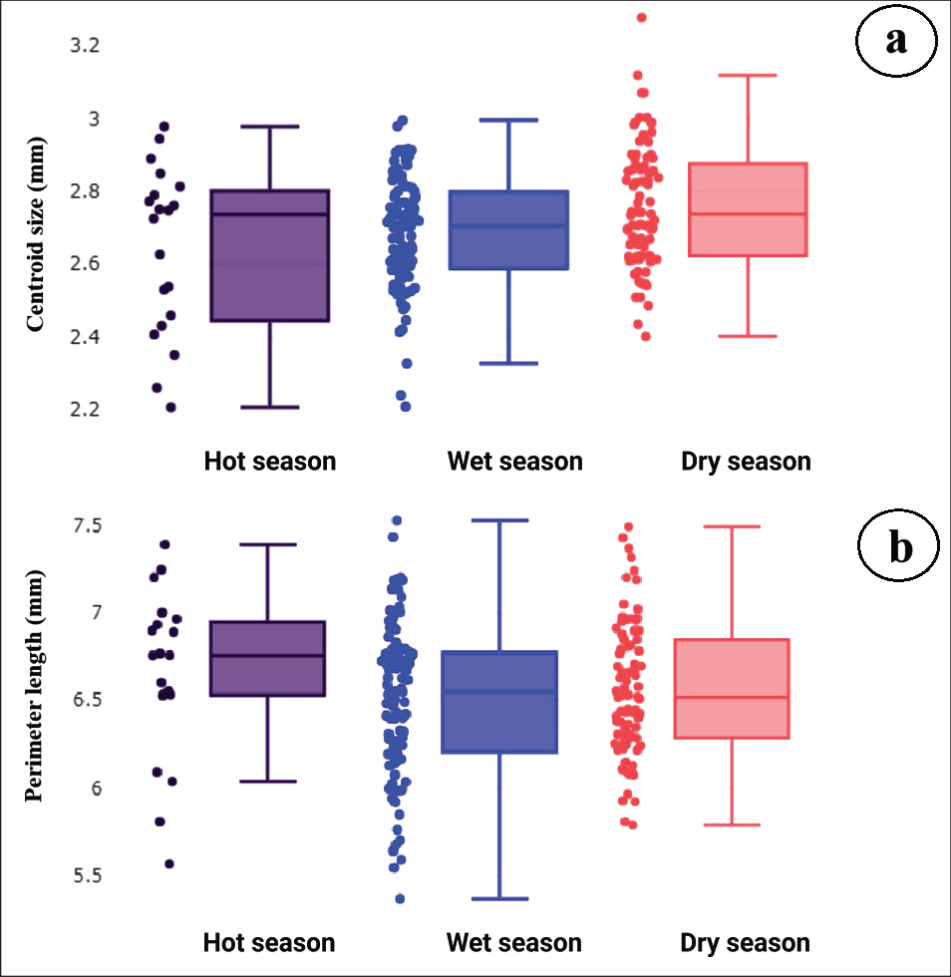
Boxplots depicting wing size variation in *Anopheles maculatus* across three seasons: (a) centroid size based on landmark-based GM analysis and (b) perimeter length based on outline-based GM analysis. Each box represents *A. maculatus* for each season and shows the group median, which separates the 25th and 75th quartiles.

Before conducting GM analyses of wing structure, the precision of digitizing landmarks was assessed. Repeatability measurements yielded satisfactory shape scores at 92%, with a measurement error of 8%, suggesting low error rates. Variation in wing CS among the *A*.* maculatus* seasonal populations is depicted in Figure 3. The wing CS ranged from 2.20 to 3.28 mm. *Anopheles* *maculatus* in the dry season exhibited the largest average wing CS at 2.76 mm, followed by the wet season at 2.69 mm and the hot season at 2.64 mm. Furthermore, a significant difference (*p *< 0.05) in wing CS was observed between the populations in the hot season and the dry season (Table 2).

**Figure 4.**
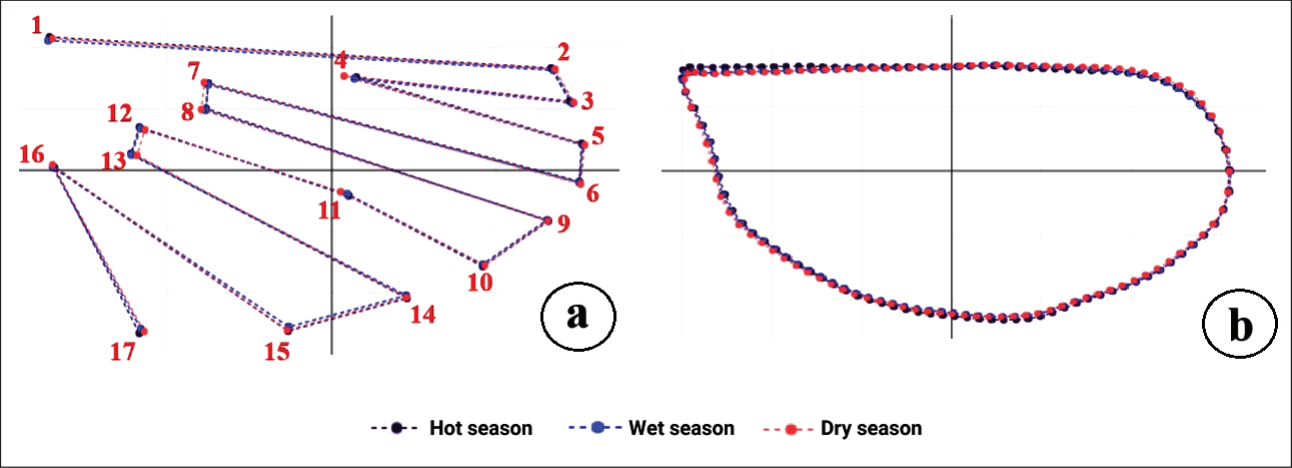
Superpositions of (a) the mean landmark configurations for wing structure based on landmark-based GM analysis and (b) the mean outlines for wing contour based on outline-based GM analysis of *Anopheles maculatus* across three seasons.

**Figure 5.**
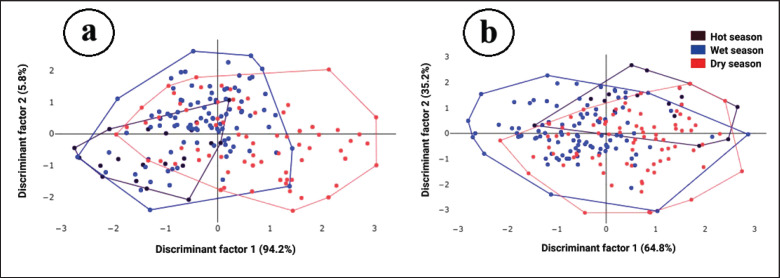
Factor maps based on wing shapes derived from discriminant analyses of *Anopheles maculatus* collected during three seasons: (a) wing structure using landmark-based GM analysis and (b) wing contour using outline-based GM analysis. Each polygon represents the shape variation within each group, with the relative contributions of each discriminant factor indicated in parentheses.

When superimposing the mean landmark configurations for wing structure of *A*. *maculatus* among seasonal populations after rotating all samples’ landmarks, differences in mean shape were observed (Fig. 4a). The factor map from the landmark-based DA of wing shape indicated overlapping distributions for all three groups (Fig. 5a). Based on pairwise comparisons of Mahalanobis distance values, significant differences (*p* < 0.05) in wing shape were found between two pairs: *A*. *maculatus* in the dry season and the hot season and *A*. *maculatus *in the dry season and the wet season (Table 3).

**Table 2.** Statistical differences in the mean wing size of *Anopheles maculatus* across three seasons.

Season	*n*	Mean (mm)	Min–Max	SD	SE
**Wing structure based on landmark-based GM analysis**					
Hot	20	2.64^a^	2.20–2.99	0.22	0.05
Wet	99	2.69^a, b^	2.21–3.00	0.15	0.02
Dry	81	2.76^b^	2.40–3.28	0.17	0.02
Wing contour based on outline-based GM analysis					
Hot	20	6.60^a^	5.53–7.30	0.47	0.10
Wet	99	6.45^a^	5.31–7.47	0.44	0.04
Dry	81	6.48^a^	5.69–7.41	0.38	0.04

Different superscript letters within an analysis indicate significant differences (*p *< 0.05) between seasons. *n* = sample size; Min = minimum; Max = maximum; SD = standard deviation; SE = standard error.

**Figure 6.**
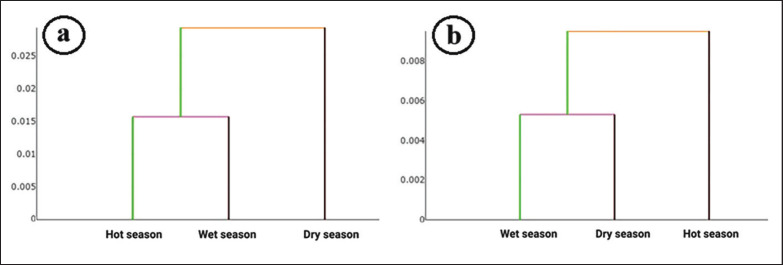
Single-linkage hierarchical classification trees for the wing shape of *Anopheles maculatus* collected during three seasons: (a) wing structure using landmark-based GM analysis and (b) wing contour using outline-based GM analysis.

The overall cross-validated classification score for the wing structure of *A*. *maculatus* among seasonal populations, based on landmark-based GM analysis, was 51%, with individual scores ranging from 35% to 55.56% (Table 4). *Anopheles* *maculatus* in the dry season achieved the highest correct classification score, while those in the hot season recorded the lowest. The single-linkage hierarchical classification tree, generated from the landmark-based GM analysis after 1,000 replicates, revealed that *A*. *maculatus* from the hot and wet seasons had more similar wing structures compared to those from the dry season (Fig. 6a).

### Seasonal variation in wing contour

The precision of the digitization of wing contours, as determined by a repeatability test, was 92% for shape, and the measurement error was 8%. Wing size variation in *A*. *maculatus* across seasons, based on the perimeter length of the wing contour derived from outline-based GM analysis, is depicted in Figure 3. The perimeter of the wing contour ranged from 5.31 to 7.47 mm. During the hot season, *A*. *maculatus* exhibited the longest average wing perimeter at 6.60 mm, with slightly shorter measurements recorded in the dry (6.48 mm) and wet (6.45 mm) seasons. However, statistical evaluation indicated that these size differences in wing contour among the seasonal groups were not significant (*p* > 0.05; Table 2).

By superimposing the mean outlines of the wing contour of *A*. *maculatus* across seasonal populations, observable differences in shape became evident (Fig. 4b). The factor map from the outline-based DA revealed overlapping distributions for all three seasonal groups (Fig. 5b). Based on pairwise Mahalanobis distances, significant differences (*p *< 0.05) in wing contours based on shape were identified between *A*. *maculatus* in the dry season and the wet season (Table 3).

The overall cross-validated classification score for *A*. *maculatus* among the seasonal populations, derived from outline-based analysis, was 49.50%, with scores ranging from 45% to 50.51% (Table 4). *Anopheles* *maculatus* in the wet season achieved the highest correct classification score, while those in the hot season had the lowest. The single-linkage hierarchical classification tree for *A*. *maculatus*, based on 1,000 replicates, showed that *A*. *maculatus* from the dry and wet seasons had more similar wing contours than those from the hot season (Fig. 6b).

## Discussion

In this study, seasonal wing variation in *A.* *maculatus*, Thailand’s principal malaria vector, was analyzed using landmark-based GM analysis for wing structure and outline-­based GM analysis for wing contour. Our results demonstrated distinct seasonal variation in wing structure and wing contour. In the size analysis, significant differences were noted between the hot and dry seasons in wing structure, while no significant differences were found across any seasonal populations in wing contour based on size. For wing shape, significant differences were observed in wing structure between the dry and hot seasons and between the dry and wet seasons. Conversely, wing contour analysis based on shape revealed a significant difference only between the dry and wet season populations. These findings clearly reflect the impact of seasons on the size and shape of *A*. *maculatus* wings from malaria hotspot villages along the Thai-Myanmar border. This aligns with recent research conducted in western Thailand, which confirmed that seasonal climatic conditions significantly influence the wing physiology of several vector mosquitoes, including *Aedes albopictus*,* Anopheles minimus*,* Armigeres subalbatus*,* Culex tritaeniorhynchus*, and *Mansonia annulifera *[28].

**Table 3.** Statistical differences in Mahalanobis distance values based on wing shapes of *Anopheles maculatus *across three seasons. Wing structure values using landmark-based GM analysis are below the diagonal, and wing contour values using outline-based GM analysis are above the diagonal.

Season	Hot	Wet	Dry
Hot	0.00	1.33	1.28
Wet	1.76	0.00	1.22*
Dry	2.56*	1.55*	0.00

Asterisks indicate significant differences (*p* < 0.05) between seasons.

**Table 4.** Cross-validated reclassification scores from two GM analyses based on wing shape similarities across three seasonal populations of *Anopheles maculatus*. The numbers within parentheses are the number of correctly assigned samples and the total observed samples, respectively.

Season	Cross-validated reclassification scores (%)	
	Wing structure using landmark-based GM analysis	Wing contour using outline-based GM analysis
Hot	35.00% (7/20)	45.00% (9/20)
Wet	50.51% (50/99)	50.51% (50/99)
Dry	55.56% (45/81)	49.38% (40/81)
Total	51.00% (102/200)	49.50% (99/200)

Based on the results of the detailed phenotypic analysis of wing size and shape in *A*.* maculatus*, it was found that wing structure was more sensitive to seasonal changes than wing contour. The landmark-based GM approach captures morphological features that are more responsive to environmental variation. While the outline-based GM approach focuses only on the outer edge of the wing, the landmark-based approach examines key anatomical intersections of the wing veins. These internal structures are critical for the wing’s function and mechanical integrity. The arrangement of wing veins plays a vital role in maintaining wing stiffness and influencing flight dynamics. Since these internal features are closely associated with physiological and functional traits, their variation is likely a result of adaptation to seasonal changes in temperature, humidity, and wind conditions [28].

Dujardin [23], Lorenz et al. [20], and Suesdek [29] have all noted that wing outlines are less informative for studying microevolution because they do not capture the internal relative positions of wing veins in sufficient detail. The greater sensitivity of wing structure to seasonal variation reinforces its importance as a morphological trait responsive to environmental changes. Accordingly, our analyses support the use of the landmark-based GM approach over the outline-based GM approach for assessing microevolutionary changes, which may enhance our understanding of evolutionary responses in *A*. *maculatus* and similar species, and guide future research in this field.

For size analysis of wing structure, *A*. *maculatus* exhibited the largest wing size in the dry season, followed by the wet and hot seasons. Large wings typically result from optimal environmental conditions in the water sources during the immature stages. Slow-flowing streams, which serve as suitable breeding sites, are more prevalent during the dry season, when the absence of strong-flowing water does not disrupt larval development [30]. Moreover, ambient temperatures crucially influence mosquito wing size [31]. Higher temperatures generally accelerate developmental and metabolic rates, leading to smaller adult body sizes, while lower temperatures slow these rates, potentially resulting in larger adult body sizes [31,32]. In western Thailand, the dry season is characterized by lower temperatures and the hot season by higher temperatures, which may explain why *A. maculatus* has the largest wings in the dry season and the smallest in the hot season, with significant differences observed in the analysis of wing structure. This phenomenon is consistent with previous studies indicating that many mosquito species, including *Anopheles epiroticus*,* Culex sitiens*,* M. annulifera*,* Mansonia indiana*, and *Mansonia uniformis*, exhibit larger wing sizes in colder conditions compared to warmer seasons [33,34]. While the size analysis of the wing outline also indicated that the dry season population had a larger mean wing size than other seasons, no significant differences with the other seasons were found. Additionally, the wet and hot seasons showed inconsistent results in the analysis of wing structure size. This suggests that the size of the wing outline may not be sensitive enough to assess seasonal environmental influences due to the lack of internal components in the analysis.

Shape analysis of the wing structures of *A*. *maculatus* showed that the dry season population differed from the populations in the other two seasons. Similarly, shape analysis of the wing contours indicated differences between the dry and wet season populations. Wing size is often considered a key factor in morphological variation, primarily because it is more influenced by environmental factors than by genetic differences. However, wing shape is also an important indicator of environmental effects [35–38]. A recent study examined phenotypic variation in a population of *Anopheles cruzii* in the southern region of São Paulo, Brazil, where the mosquitoes inhabited an urban environment. The study found a strong association between wing shape and the natural environmental conditions in which the mosquitoes lived [39]. In Thailand, wing shape differences have also been reported in several mosquito species, including *A*. *albopictus* [40], *M*. *uniformis* [41], and *Culex gelidus* [42]. These differences are attributed to the varying environmental conditions in each area, suggesting that environmental factors have an important effect on determining wing morphology and influencing physiological adaptations.

Additionally, temperature differences may be the primary factor driving seasonal changes in mosquito wing shape. In 2015, weather data from Mae Sot, Tak Province, Thailand, showed clear seasonal temperature variation. The average minimum temperature was 25.37°C during the dry season, 26.63°C in the wet season, and 28.87°C in the hot season. A recent study used the landmark-based GM method to investigate how larval temperature affects the wings of *A. albopictus* [43]. The study found that lower temperatures significantly influenced the wing shape of female mosquitoes from the field strain. These findings are consistent with the current study, which observed seasonal variation in wing shape in *A*. *maculatus*, particularly during cooler periods. For instance, the larger wing structures seen during the dry season may enhance mosquito flight capacity and dispersal, enabling them to travel farther in search of hosts or breeding sites. Understanding how environmental factors affect wing shape can inform more strategic vector control planning. For example, targeted interventions such as distributing long-lasting insecticidal nets could be timed to periods when mosquitoes are more likely to disperse. These insights provide a strong foundation for developing vector control strategies that align with seasonal changes in local mosquito populations.

## Conclusion

In this study, we used landmark-based GM analysis to examine the structure of *A. maculatus* wings and outline-based GM analysis to assess wing shape. *Anopheles* *maculatus* is the primary malaria vector in Thailand. The results showed that wing structure varied more across seasons than wing contour. This is likely because the landmark-based GM approach is more effective at capturing distinct features of the wings. Therefore, the landmark-based method is more suitable than the outline-based method for detecting subtle evolutionary changes. The study demonstrates that seasonal environmental factors significantly influence the shape of *A*.* maculatus* wings. Wings were larger during the cooler dry season and smaller during the hotter season. Additionally, wing shape serves as an important indicator of environmental influence, with dry season temperatures having a particularly strong impact. These findings are valuable for understanding how this mosquito vector adapts to seasonal changes. Incorporating morphometric data into routine entomological surveillance could provide an early-warning system for shifts in vector populations driven by environmental changes, thereby enabling proactive and adaptive public health responses. Future research should investigate the evolutionary mechanisms underlying these adaptations and determine whether similar seasonal patterns occur in other malaria vectors across diverse regions.

## List of Abbreviations

ANOVA, analysis of variance; CDC, Centers for Disease Control and Prevention; CS, Centroid size; DA, discriminant analysis; DF, discriminant factor; GM, geometric morphometrics; LLINs, Long-lasting insecticidal nets; NEF, Normalized Elliptic Fourier; SD, standard deviation; SE, standard error; XYOM, XY Online Morphometrics tool; °C, degree Celsius; mm, millimeter; n, sample size.
